# Antimicrobial Peptides as New Combination Agents in Cancer Therapeutics: A Promising Protocol against HT-29 Tumoral Spheroids

**DOI:** 10.3390/ijms21186964

**Published:** 2020-09-22

**Authors:** Mina Raileanu, Aurel Popescu, Mihaela Bacalum

**Affiliations:** 1 Department of Electricity, Solid State and Biophysics, Faculty of Physics, University of Bucharest, 077125 Măgurele, Romania; mina.raileanu@nipne.ro (M.R.); prof.aurel.popescu@gmail.com (A.P.); 2 Department of Life and Environmental Physics, Horia Hulubei National Institute in Physics and Nuclear Engineering, 30 Reactorului Str., 077125 Magurele, Romania

**Keywords:** antimicrobial peptides, doxorubicin, 3D tumoral spheroids, gramicidin A

## Abstract

Antimicrobial peptides are molecules synthetized by a large variety of organisms as an innate defense against pathogens. These natural compounds have been identified as promising alternatives to widely used molecules to treat infections and cancer cells. Antimicrobial peptides could be viewed as future chemotherapeutic alternatives, having the advantage of low propensity to drug resistance. In this study, we evaluated the efficiency of the antimicrobial peptide gramicidin A (GA) and the anticancer drug, doxorubicin (Doxo) against the spheroids from colorectal cancer cells (HT-29). The two drugs were applied separately against HT-29 spheroids as well as together to determine if they can act synergistically. The spheroid evolution, cell viability, and ATP levels were monitored at 24 and 48 h after the applied treatments. The results show significant drops in cell viability and cellular ATP levels for all the experimental treatments. The simultaneous use of the two compounds (GA and Doxo) seems to cause a synergistic effect against the spheroids.

## 1. Introduction

Despite recent advances in medical treatment, cancer still remains a worldwide leading cause of death. Therapy based on surgery, radiotherapy, chemotherapy, or a combination of these can extend the patient survival period. However, there are many obstacles that can influence or limit their efficiency. Some of the obstacles are due to intratumor complexity and heterogeneity and to cell interactions inside the tumor or with the surrounding microenvironment [1,2]. These can limit the drug access to the whole tumor volume, leading to chemotherapy resistance [1,3,4]. Another unpleasant problem comes from the lack of specificity of some of the anticancer drugs, which also kill healthy cells, resulting in toxic side effects [5]. 

In recent years, some of the attractive compounds that have been tested in vitro, which are expected to surpass the conventional drug limitations, are the antimicrobial–anticancer peptides (AMPs). AMPs are essential components of the host innate immunity system, which are secreted by a large number of organisms as a response to various pathogens and stress conditions [6]. Due to their characteristics, such as high hydrophobicity and positive net charge, AMPs could be considered a precious resource, with low propensity to trigger the development of cancer cell resistance. These characteristics give the peptides an increased affinity for cell membranes. Compared to the healthy cell membranes, cancer cell membranes are negatively charged, facilitating the attachment of the peptide to the membrane, followed by its disruption through different mechanisms. Moreover, AMPs have a reduced toxicity to rapid proliferating healthy cells [7,8,9,10]. Depending on the peptide structure and concentration, different AMP action mechanisms have been described, such as the formation of membrane detergent-like “carpets” or of discrete membrane pores that dissipate the ion gradients [11,12,13]. 

Until now, most of the results concerning the anticancer effects of AMPs were obtained from experiments performed in two-dimensional (2D) cell cultures [14,15]. In 2D cultures, the cells are grown under unrealistic conditions on rigid materials, such as polystyrene and glass. This type of culture does not correctly reflect the true morphology of the real tissues. These conditions modify the tissue-specific architecture (3D cell shape), mechanical and biochemical signals, and subsequent cell-to-cell communication pathways, all influencing the results [16]. These limitations are overcome by using 3D culture systems (i.e., spheroids). Cellular spheroids more accurately mimicked some of the tumor features [17], thus becoming important tools for in vitro studies related to drug delivery and antidrug resistance [18]. 

Due to spheroid complexity, it is expected that the AMP concentrations needed to exert the same biological effect as in 2D culture will be higher, similar to the other studied drugs [19,20]. An alternative promising approach reported by the scientific community is the use of drug combination protocols, which give better results in spheroids or tumor xenografts [21], as well as in patients [22].

A peptide that has received great interest in recent years is gramicidin A (GA), a 15 aa short peptide produced by *Aneurinibacillus migulanus* (formerly known as *Bacillus brevis*), with known efficiency against bacteria, fungi, and protozoa [23,24]. GA is known to form ion channels, which can allow monovalent ion (Na^+^ and K^+^) diffusion, resulting in changes in intracellular osmolality, and finally in cell death [25]. Recently, its antitumoral effect on renal cell carcinoma [26,27], gastric cancer cells [28], or pancreatic cells [29] was reported. Moreover, it was shown that GA used in combination with curcumin kills the cells expressing the multidrug-resistance-linked ABCG2 transporter by stimulated depletion of ATP levels [30].

The aim of this study was to evaluate the effects of two compounds—GA (an AMP) and a known anticancer drug, Doxo—delivered separately or in combination against 3D cell culture systems (i.e., spheroids). The experiments were performed on HT-29 colorectal cancer cells and the spheroid evolution for all experimental conditions was evaluated by light microscopy. Cell viability and ATP assays were used to quantify the GA and Doxo effects on the investigated systems. Both compounds, delivered separately or in combinations, affect the spheroid organization, decreasing the cell viability and cellular ATP level. It was shown that the two compounds act synergistically against the 3D colorectal cancer cells.

## 2. Results

### 2.1. HT-29 Spheroids Evolution

**Figure 1:**
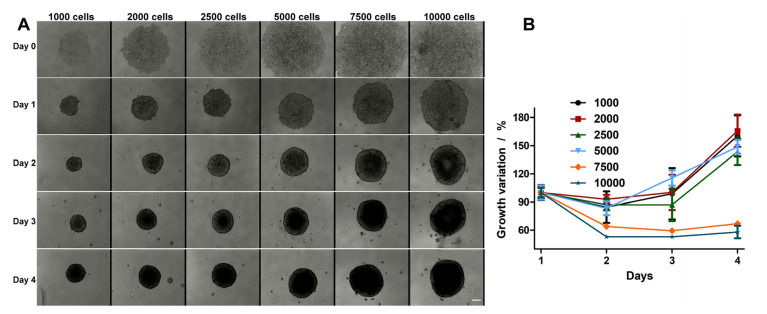
(**A**) Spheroid evolution monitored over 5 days at different seeding densities (1000, 2000, 2500, 5000, 7500, and 10,000 cells/well). (**B**) Growth kinetics of HT-29 spheroids. The scale bar is 100 μm and is the same for all images.

HT-29 spheroid formation was evaluated for different cell densities. In Figure 1A, the representative images for each condition evaluated over 5 days are shown, starting from the day of seeding onto the plates (day 0) and finishing on the fifth day (day 4). As one can observe, on the first day, the cells do not form spheroids and are still being dispersed in the well. However, after 24 h (day 1), one can see that the formed spheroids have a well-defined border. The bonds between cells are even more stabilized after 48 h (day 2), when one can see more compacted spheroids with a denser core and better defined edges.

In order to assess the growing curve of each experimental condition and to select the appropriate condition for the experiments, the growth variations of the spheroids were recorded and compared. Based on the curves presented in Figure 1B, we found that the spheroids, obtained at densities ranging from 7500 and 10,000 cells/well, were formed on day 2, but over the following two days do not significantly increase. For the densities of 1000 and 2500 cells/well, only on the 4th day can the evolution of the spheroid size be seen. The best growth curve was obtained for the spheroids formed by starting with 5000 cells/well. After 48 h and over the following days, the spheroid size was well formed and constantly increased (Figure 1B). For this reason, all the following experiments were performed on spheroids prepared from 5000 cells/well, with the treatment administered on the second day after plating.

### 2.2. Treatment Effect on HT-29 Spheroid Morphology

**Figure 2:**
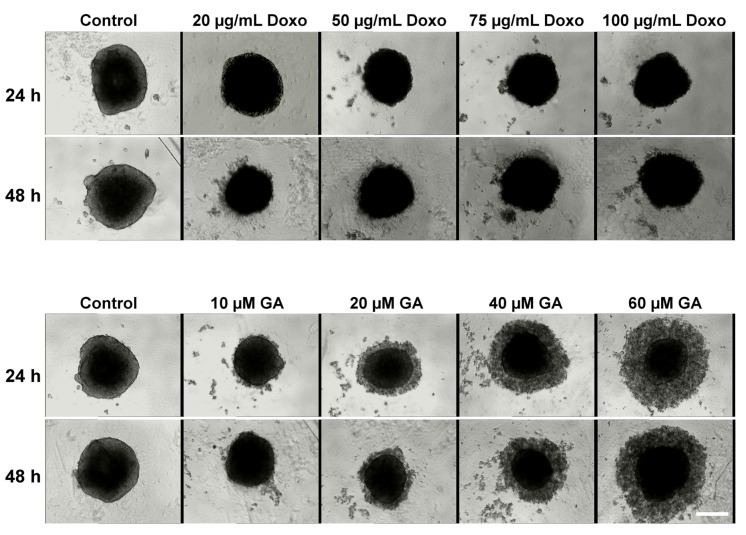
Cytotoxic effects of doxorubicin (Doxo) and gramicidin A (GA) on HT-29 spheroids. Spheroids were obtained after seeding 5000 cells/well and were grown for three days. On the third day of spheroid growth, different concentrations of Doxo (20, 50, 75, and 100 μg/mL) and GA (10, 20, 40, and 60 μM) were added and the effects were monitored through transmission microscopy at 24 and 48 h. The scale bar is 200 μm and is the same for all images.

**Figure 3:**
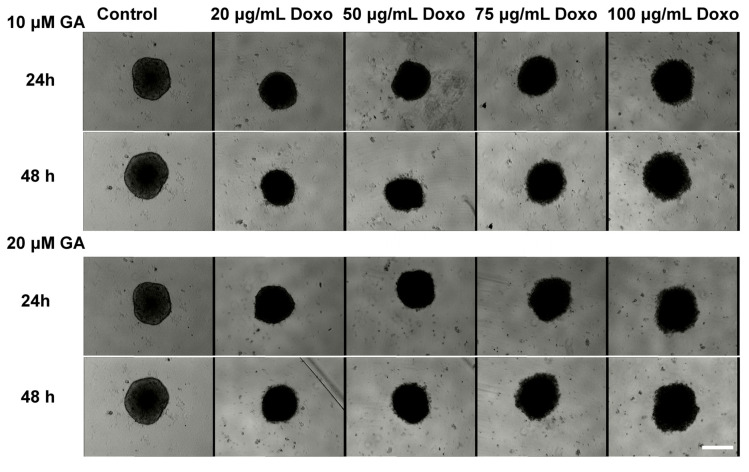
Cytotoxic effects of Doxo in combination with GA on HT-29 spheroids. Spheroids were obtained after seeding 5000 cells/well and were grown for three days. On the third day of spheroid growth, different concentrations of Doxo (20, 50, 75, and 100 μg/mL) and GA (10, 20, 40, and 60 μM) were added and the effects were monitored through transmission microscopy at 24 and 48 h. The scale bar is 200 μm and is the same for all images.

**Figure 4:**
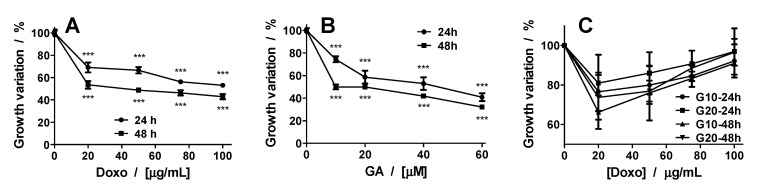
Growth variation of spheroids treated with Doxo (**A**), GA (**B**), and their combination (**C**). The *p* values were based on ANOVA analysis with Bonferroni’s multiple comparison post-test applied. (**C**) GA10-24 h: Control vs. 20 μM and 50 μM**, 75 μM*, and 100 μM (ns). GA20-24 h: Control vs. all concentrations—ns. GA10-48 h: Control vs. 20 μM***, 50 μM**, 75 μM*, and 100 μM (ns). GA20-48 h: Control vs. 20 μM and 50 μM***, 75 and 100 μM (ns). Note: * *p* < 0.05, ** *p* < 0.01, *** *p* < 0.001; ns—not significant.

The spheroid morphology after treatment with GA, Doxo, and their combination was monitored by transmission light microscopy (Figure 2 and Figure 3). The growth variation is presented in Figure 4. In the case of the Doxo treatment, one can see that the sizes of the spheroids decrease both with increasing concentration and treatment time (Figure 2 and Figure 4A).

The treatment does not destabilize the spheroids, but makes them darker (Figure 2). Compared with the control spheroids, which show a darkening of the core, for Doxo-treated spheroids, one can see a darkening of the entire spheroid. On the contrary, for GA treatment, starting with the second tested concentration (20 μM), the spheroids begin to destabilize and dead cells start to detach from them. When representing the growth curves, only the compact core of the spheroid was considered. The growth curves were similar to those obtained for Doxo treatment (Figure 4A,B).

The effects induced by the mixed treatment were assessed for the combination of GA (10 μM and 20 μM) with Doxo in increasing concentrations (Figure 3). For the combined treatment, the spheroids decreased in size with the smallest concentration of Doxo. With increasing concentration of GA in the presence of Doxo, the spheroid sizes increased and the spheroids became darker (Figure 4C).

### 2.3. Synergetic Effect of Doxo and GA Treatments on HT-29 Spheroids

**Figure 5:**
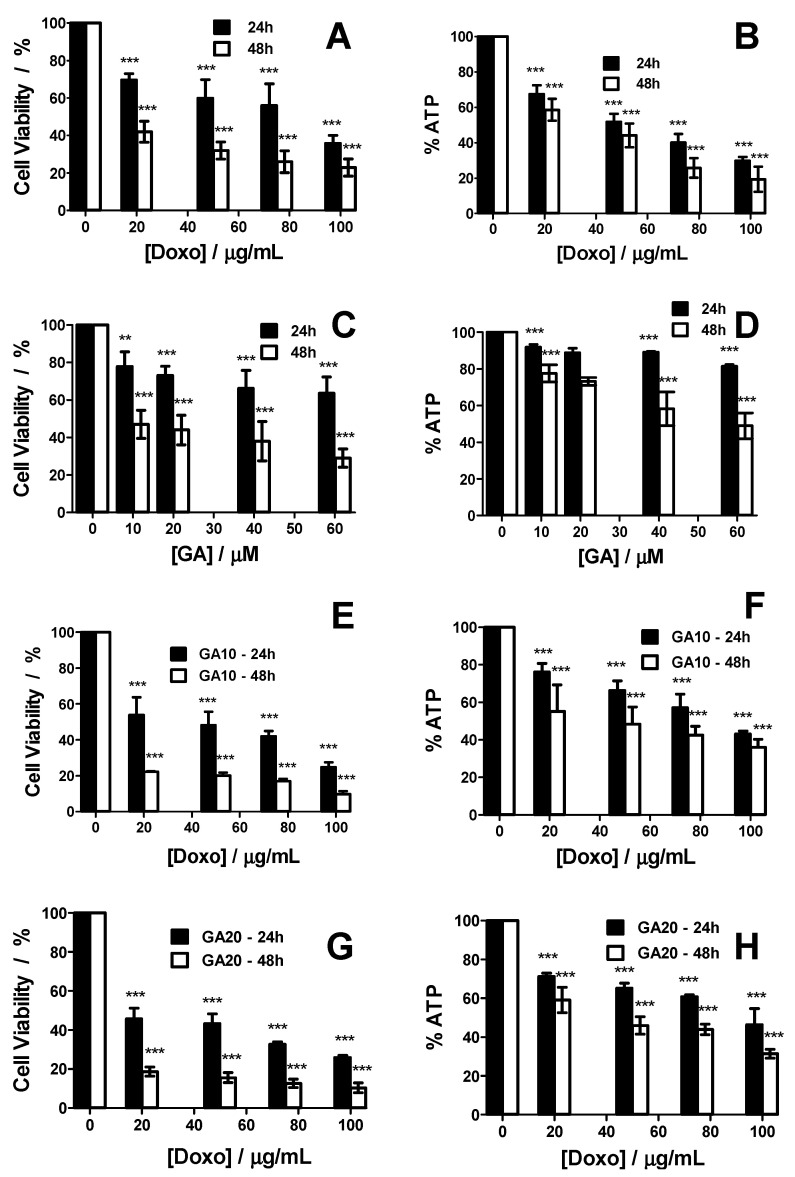
Cell viability of HT-29 spheroids treated with Doxo (**A**), GA (**C**), and their combination (**E**,**G**) at 24 and 48 h with increasing concentrations. ATP levels of HT-29 spheroids treated with Doxo (**B**), GA (**D**), and their combination (**F**,**H**) at 24 and 48 h with increasing concentrations. *p* values based on ANOVA analysis with Bonferroni’s comparison post-test versus control condition. Note: ** *p* < 0.01, *** *p* < 0.001.

The viability of HT-29 3D culture treated with various concentrations of GA or Doxo was evaluated by MTT assay at 24 and 48 h, the results of which are presented in Figure 5A,C. Both compounds when used separately significantly decreased the viability of HT-29 spheroids in a monotonous mode with increasing concentrations. Compared to 24 h, after 48 h of treatment, the drop in cell viability was significantly higher. The results reveal that the two compounds have a cytotoxic effect against the HT-29 spheroids. Whenever possible, the half-maximal inhibitory concentration (IC_50_) values were estimated using a dose–response function to fit the data. Thus, for Doxo, the IC_50_ values are 68.72 μg/mL at 24 h and 15.31 μg/mL at 48 h, while for GA at 48 h only, an IC_50_ of 9.78 μM was found.

Besides the cell viability, the cellular ATP levels of spheroid cells were measured (Figure 5B,D). In this respect, a higher decrease of ATP was observed in spheroids treated with Doxo than in spheroids treated with GA. Similar to cell viability, a higher effect was observed at 48 h as compared to that at 24 h for the cellular ATP levels.

In addition to using the two drugs separately, we combined them to see if they could have a possible synergistic effect (Figure 5E–H). We used two different GA concentrations (10 and 20 μM), each in combination with the same concentration of Doxo used alone (0 to 100 μg/mL). The cell viability was significantly lower with combined treatment protocol, with higher losses of viability at 24 and 48 h as compared to the two drugs separately administered.

The ATP level after using both drugs in combination loered with increasing concentration, but not as much as in the case of separately applied Doxo. There was also a clear difference when the combined treatment was applied for 24 or 48 h.

**Figure 6:**
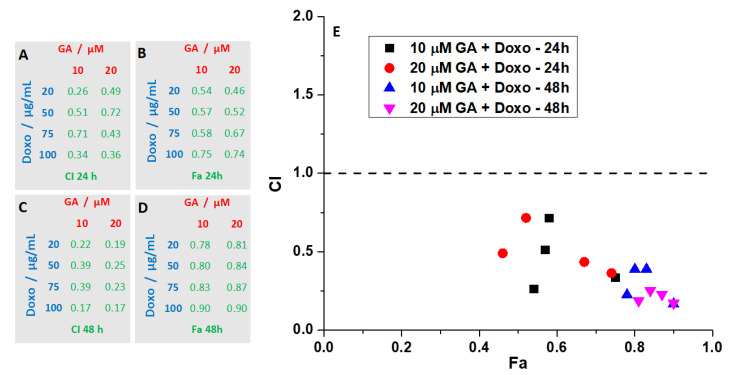
Effects of combination therapy of GA and Doxo on HT-29 spheroids. (**A**,**C**) Combination index (CI) values at the concentrations tested. (**B**,**D**) Fraction affected (Fa) values at the concentrations tested. (**E**) The CI versus Fa values, plotted for different conditions, indicate synergism for all tested conditions. CI < 1 indicates synergism, CI = 1 additivity, whereas CI > 1 indicates antagonism.

Both GA and Doxo showed good results when used separately, but in combination, higher concentrations were needed. Due to its action mechanism (i.e., forming pores in the plasma membranes of the cells), GA showed a significant response from the first concentration tested (10 μM), followed by lesser effects with increasing concentrations. As for Doxo, the decrease of viability was monotonous with increasing concentrations. Based on the results reported before, we decided to use only the two smallest concentrations of GA, together with different concentrations of Doxo. This type of protocol, where one of the drugs has a fixed concentration and the concentration of the second one is varied, has been applied successfully for different drug combinations, as reported in the literature [31,32,33,34]. Furthermore, in order to assess if the combination of the two drugs has a synergistic effect, we calculated the combination index (CI) and found that all conditions tested were synergistic (CI < 1). In Figure 6, the CI as a function of the fraction affected (Fa) for all tested combinations is reported. CI values < 1 are indicative of a synergistic effect. The best results were found at 48 h for the combination treatment using 20 μM of GA.

## 3. Discussion

Recent studies have linked the microenvironment present in tumors to the coordination of tumor growth, metastasis, and resistance to anticancer therapies [35]. Additionally, the fact that 2D systems do not correctly predict a drug’s therapeutic efficacy in viv has led to the increase usage of 3D spheroid tumor models for drug evaluation, which better mimic in viv conditions of the intratumoral space [36].

Colorectal cancer has a wide distribution globally, and is reported to be the second most common cancer for women and the third for men [37,38]. The efficiency of colorectal cancer therapy and patient survival are limited by side-effects, such as the development of drug resistance or high toxicity to healthy cells [39,40]. Finding new, more efficient drugs [41] or using combination chemotherapy [39,40] are some of the solutions to the mentioned problems. Recent studies have reported the use of various drugs (5-Fluorouracil, Simvastatin, and Irinotecan) or natural compounds (gelam honey, curcumin, etc.) as possible synergistic combinations against colon cancer cell lines in vitro and in vivo [42,43,44,45,46]. Taking into account the recent momentum around the use of anticancer peptides, there are a significant number of studies on drugs that are more efficient against colon cancer cell lines [14,15,47,48,49,50]. However, there are only a few studies where peptides are tested on colon cancer spheroids [51,52,53].

Considering these factors, the combination of conventional chemotherapeutic agents with natural compounds, in our case an AMP (GA), can be seen as a new approach with possibly better impacts on colon cancer research and treatment.

As already mentioned, we have studied the effects of GA and a known chemotherapeutic drug Doxo, administered separately and in combination, against HT-29 colorectal spheroids. The two molecules were selected due to their different action mechanisms—GA is an ionophore that forms membrane channels and reduces ATP levels in the cells [26,54], while Doxo is known to generate reactive oxygen species, altering DNA and impairing DNA repair processes [55]. When selecting the drugs, we also considered their molecular size. Previous studies have shown that using two anticancer compounds with different molecular weights reduces the penetration efficiency of the high molecular weight agents [56]. In our case, by using the two compounds, both with low molecular weights, we showed that either separate or combination treatments are efficient against the spheroids. When treated with only one of the drugs, the spheroid diameters decreased, with GA having the biggest impact. At higher concentrations of GA, the spheroids were destabilized, resulting in a cloud of detached cells around the main core of the spheroids. On the contrary, in the case of Doxo treatment, the spheroids remained intact, but became darker with increasing drug concentrations. However, when used in combination, the two drugs induced an increase in spheroid size. This could be due to GA, which when used in small concentrations led to an alteration of the spheroid integrity, allowing better penetration of Doxo, as observed by the darkening of the spheroids, even at smaller concentrations. 

The monitoring of cell viability by analyzing the spheroid morphology confirmed the results—both drugs applied separately are efficient against the spheroids, while their combination decreases the cell viability even more. Due to its action mechanism, Doxo is more efficient after 24 h as compared to GA. However, GA’s effect is better after 48 h. This could be due to Doxo’s penetration efficiency, which decreases over time. However, GA can destabilize the integrity of the spheroids, and a longer time is needed to see the effects. The two different action mechanisms become important when the two drugs are used together. The GA facilitates better penetration of the drug inside the spheroids, as observed from the lower viability curves obtained after combined action of the two molecules.

When cellular ATP levels were measured for both molecules separately, the ATP levels decreased with concentration and time. A greater decrease of ATP levels was found in Doxo-treated spheroids as compared with the ones treated only with GA. This finding for GA was an expected result based on previous studies, which showed that this peptide induces energy depletion in renal cell carcinoma [26,27].

Furthermore, Doxo is known to kill colon cancer cells through apoptosis [57], and it was shown in other cell lines to inhibit cellular respiration, leading to increased toxicity and decreased ATP levels [58,59]. Therefore, the results found for the two drugs when administered separately were expected. However, a surprising effect was found when the two drugs were used in combinations. Although we expected a higher energy depletion, the experimental data showed a higher cellular ATP level as compared to Doxo alone, suggesting that GA protects the cells against Doxo treatment. Although the protective effect against Doxo was reported in a previous study [58], the objectives of the mentioned study was not to find the mechanisms by which energy depletion is induced by the two drugs separately or in combination.

Based on previous results, Doxo showed a synergistic effect in combination with other molecules against various cell lines, both in vitro and in vivo [60,61,62,63].

Ultimately, we checked via the combination index (CI) if the two tested compounds have a synergistic effect. Using the CI, one could also determine the type of interaction [64]. The GA and Doxo combination at 24 h generated CI values ranging between 0.25 and 0.71, indicating that the type of interaction is synergistic. For the same combination at 48 h, the CI values varied between 0.16 and 0.38, indicating strong synergism.

## 4. Materials and Methods

### 4.1. Materials

Doxorubicin (Doxo) and gramicidin A (GA) were purchased from Sigma-Aldrich (Saint Louis, MO, USA). GA’s structure was HCO-L-Val-Gly-L-Ala-D-Leu-L-Ala-D-Val-L-Val-D-Val-L-Trp-D-Leu-L-Trp-D-Leu-L-Trp-D-Leu-L-Trp-NHCH_2_CH_2_OH. Dimethyl sulfoxide (DMSO) was purchased from Merck (Darmstadt, Germany), 3-(4,5-dimethylthiazol-2-yl)-2,5-diphenyltetrazolium bromide (MTT) was purchased from Serva (Heidelberg, Germany) and CellTiter-Glo^®^ Luminescent Cell Viability Assay was purchased from Promega Corporation (Fitchburg, WI, USA). All cell cultivation media and reagents were purchased from Biochrome AG (Berlin, Germany).

### 4.2. Cell Culture

Human colorectal adenocarcinoma HT-29 cells (ATCC, Manassas, VA, USA) were cultured in minimum essential medium (MEM) supplemented with 10 % fetal bovine serum (FBS) and penicillin–streptomycin (0.05%—100 units/mL) in a humidified incubator under an atmosphere containing 5% CO_2_.

### 4.3. Spheroid Formation and Analysis

Different seeding concentrations of HT-29 cells (1000, 2000, 2500, 5000, 7500 and 10,000 cells/well) were used to evaluate spheroid formation over 5 days. A final volume of 200 µL of cell suspension was placed in each well of a clear, round bottom, ultra-low attachment 96-well microplate (Corning, NY, USA). After this, the plate was centrifuged for 2 min and then incubated at 37 °C for up to 5 days. Spheroid formation was confirmed by observing the plate under a light microscope (Olympus CX23 Binocular Microscope, Düsseldorf, Germany). Spheroids were monitored daily and the incubation medium was replaced every 3 days.

### 4.4. Treatment of HT-29 Spheroids

Treatment evaluation was performed on spheroids obtained from an initial suspension of 5000 cells/well. After 3 days, the treatments with the Doxo and GA, either separately or in combination, were applied. Doxo alone was added in four concentrations (20, 50, 75, and 100 μg/mL), while GA alone was also added in four concentrations (10, 20, 40, and 60 μM). For the combined treatment, we two different GA concentrations were used (10 and 20 μM), each in combination with the other 4 Doxo concentrations. The changes in spheroid integrity were evaluated by light microscopy 24 and 48 h after treatment.

### 4.5. Cell Viability Assays

Cell viability was assessed using the MTT assay. The culture medium was removed from each well after desired treatment times (24 and 48 h). MTT was added to each well at a final concentration of 1 mg/mL and the cell culture was further incubated. After 4 h, the medium was removed and DMSO was added to dissolve the crystals that had formed. Optical absorbance was recorded at λ = 490 nm using a Mithras LB 940 plate reader (Berthold, Germany). Cell viability was calculated using the following formula:

(1) $$\%\;\mathrm{viable}\;\mathrm{cells}\;=\;\frac{\mathrm{Corrected}\;\mathrm{absorbance}\;\mathrm{of}\;\mathrm{treated}\;\mathrm{cells}}{\mathrm{Corrected}\;\mathrm{absorbance}\;\mathrm{of}\;\mathrm{control}\;\mathrm{cells}}\times 100$$

The half-maximal inhibitory concentration (IC_50_) values were estimated by fitting the data with a logistical sigmoidal equation using Origin 8.1 software (Microcal Inc., Northampton, MA, USA).

### 4.6. ATP Assays

ATP levels in the treated spheroids were assessed, as will be described below. Here, 100 μL of medium was removed from each well, then the remaining 100 μL with the spheroid was transferred into an opaque 96-well plate. After this, 100 μL of CellTiter-Glo^®^ reagent (Promega, Madison, WI, USA) was added onto the spheroids, which were incubated at room temperature for 10–15 min under thorough shaking to make sure that the spheroids were broken. Finally, the luminescence of the cells was measured using the plate reader. The percentage of ATP level was estimated using the following formula:

(2) $$\%\;\mathrm{ATP}\;=\;\frac{\mathrm{Corrected}\;\mathrm{luminescence}\;\mathrm{of}\;\mathrm{treated}\;\mathrm{cells}}{\mathrm{Corrected}\;\mathrm{luminescence}\;\mathrm{of}\;\mathrm{control}\;\mathrm{cells}}\times 100$$

### 4.7. Analysis of Combination Index (CI)

The drug–drug interactions between GA and Doxo were evaluated using the combination index (CI) described by Chou and Talalay, and calculated using Compusyn software [64,65]. CI > 1 indicates drug antagonism, CI = 1 indicates a drug additive effect, while CI < 1 indicates drug synergism.

### 4.8. Statistical Analysis

Each experiment was performed at least three times with at least 6 spheroids per condition, per experiment. All data are presented as means ± standard deviations, if not stated otherwise. The statistical analysis was carried out using the GraphPad Prism 5 software package (San Diego, CA, USA). One-way analysis of variance (ANOVA) was used to calculate statistical significance. A value of *p* < 0.05 was chosen to indicate that the difference is statistically significant.

## 5. Conclusions

In vitro experiments on three-dimensional cell cultures (i.e., spheroids) are more realistic models for the study of the drug effects as compared to two-dimensional models. The peptide gramicidin A and the drug doxorubicin administered separately can be used successfully as chemotherapeutic agents against HT-29 colon spheroids, reducing cell viability, as well as depleting cellular energy (i.e., the ATP level). Gramicidin A and doxorubicin administered simultaneously to HT-29 spheroids had a strong synergistic effect when applied for 48 h, as the combination index proved.

Although more studies are needed to better understand the mechanisms of drug synergy, our data demonstrate that this approach may become a valid strategy in treating cancer, at least in for HT-29 cells. Based on these findings, we can further say that AMPs are good candidates for future anticancer applications and in vivo studies, even in clinical trials.

## Abbreviations

CI	Combination Index
Doxo	Doxorubicin
GA	Gramicidin A
Fa	Fraction affected
